# Rapidly Growing Pancreatic Adenocarcinoma Presenting as an Irreducible Umbilical Hernia

**DOI:** 10.1155/2018/1784548

**Published:** 2018-06-13

**Authors:** Deepti M. Reddi, Kathryn P. Scherpelz, Angelica Lerma, Jabi Shriki, Jeffrey Virgin

**Affiliations:** ^1^Department of Pathology, University of Washington Medical Center, 1959 N.E. Pacific Street, Box 356100, Room NE110, Seattle, WA 98195-6100, USA; ^2^Department of Pathology, Veteran Affairs Puget Sound Health Care System, 1660 S. Columbian Way, Seattle, WA 98108, USA; ^3^Department of Radiology, Veteran Affairs Puget Sound Health Care System, 1660 S. Columbian Way, Seattle, WA 98108, USA

## Abstract

Hernia sacs are a common anatomic pathology specimen, which rarely contain malignancy. We present a case of rapidly growing pancreatic adenocarcinoma, which initially presented as metastasis to an umbilical hernia sac. The patient was a 55-year-old male with a two-year history of umbilical hernia. Two months prior to herniorrhaphy, the hernia became painful and the patient experienced nausea and weight loss. The gross examination did not reveal distinct lesions. Microscopically, the hernia sac was diffusely infiltrated by moderately differentiated adenocarcinoma, which was positive for CK7 and pancytokeratin and negative for TTF-1, CK20, PSA, and CDX2. Clinical laboratory tests found elevated levels of CA 19-9 and CEA. Computed tomography scan with intravenous contrast showed a 5 cm ill-defined and hypoattenuating mass involving the pancreatic tail and body, as well as numerous ill-defined lesions in the liver and peritoneal carcinomatosis. The patient had an earlier noncontrast computed tomography scan four months prior to the surgery, which did not detect any lesions in the abdomen. This case highlights the importance of intravenous contrast with computed tomography for the evaluation of pancreatic lesions and also emphasizes the importance of thorough histologic evaluation of hernia sacs for the detection of occult malignancy.

## 1. Introduction

Hernias are very common conditions encountered in medicine, and more than 20 million hernias are estimated to be repaired every year around the world. In the recent years, the number of midline abdominal wall hernia repairs has increased. The current relative order of the various hernia repair types is as follows in decreasing frequency: inguinal, umbilical, epigastric, incisional, paraumbilical, femoral, and rare forms, for example, spigelian [1]. We report a case of an irreducible umbilical hernia, with no macroscopic sign of malignancy during the surgery, which was diagnosed with metastatic pancreatic adenocarcinoma on routine histologic evaluation of the hernia sac.

## 2. Case Presentation

The patient is a 55-year-old man with past medical history significant for two-year history of umbilical hernia, diabetes mellitus type 2, hypertension, gout, chronic kidney disease with proteinuria, diverticulosis, obesity, and osteoarthritis. The patient presented to the clinic because of umbilical hernia pain, which developed over the two months. The pain localized to the periumbilical region and left lower back, and it was exacerbated with food intake and sometimes relieved by 5 mg hydrocodone tablet. He also reported nausea and fifteen pounds weight loss over the two months, which he attributed to decreased food intake. The physical examination showed a 1 cm tender and irreducible mass superior to the umbilicus. The patient underwent herniorrhaphy and the gross examination of the surgical specimen did not reveal any masses or lesions.

**Figure 1:**
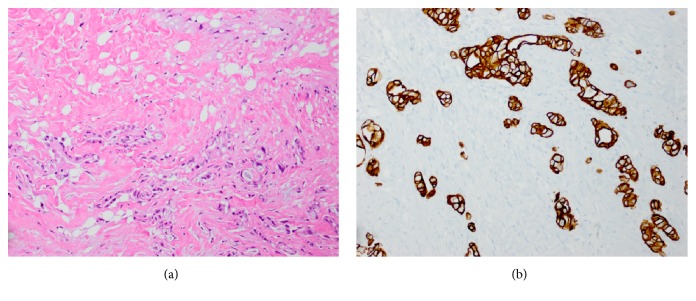
(a) Diffuse infiltration of the connective tissue by neoplastic cells with hyperchromatic nuclei, inconspicuous nucleoli, and abundant eosinophilic cytoplasm (hematoxylin-eosin, original magnifications: x200). (b) Neoplastic cells are strongly positive for CK7 (immunohistochemistry, original magnifications: x200).

The microscopic evaluation showed diffuse infiltration of the connective tissue by malignant cells with hyperchromatic nuclei, inconspicuous nucleoli, and abundant eosinophilic cytoplasm (Figure 1(a)). There were focal areas of gland formation with mucin production, consistent with adenocarcinoma. By immunohistochemistry, the neoplastic cells were strongly positive for pancytokeratin and CK7 (Figure 1(b)) and negative for CK20, CDX2, TTF-1 and PSA.

**Figure 2:**
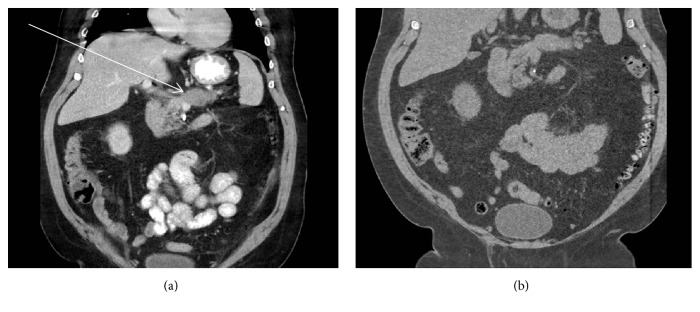
(a) Computed tomography scan with intravenous contrast, which showed 5.0 × 2.7 cm irregular, ill-defined, and hypoattenuating mass located in the pancreatic tail and body (labelled as thin arrow). (b) The prior noncontrast computed tomography scan four months earlier showed no lesions in the pancreas and abdomen.

The laboratory findings showed elevated levels of CA 19-9 (16,590 U/mL) and CEA (14.2 ng/mL). The patient underwent a subsequent computed tomography scan with intravenous contrast, which showed a 5.0 × 2.7 cm ill-defined and hypoattenuating mass located in the pancreatic tail and body (Figure 2(a)), with peripancreatic fat infiltration and vascular involvement of splenic artery and vein. In addition, the imaging showed peritoneal carcinomatosis, multiple ill-defined hypoattenuating lesions in the liver, and enlarged and hypoattenuating pericecal iliac lymph nodes. The patient had a prior noncontrast computed tomography scan four months earlier, which showed umbilical hernia with fat and no other lesions in the pancreas and abdomen (Figure 2(b)).

After the diagnosis, the patient refused chemotherapy and decided to undergo palliative care. The patient had rapid progression of the disease and died within two months of the initial histologic diagnosis of malignancy.

## 3. Discussion

Malignant tumors of the umbilical region can be primary or secondary, constituting 17% and 83%, respectively, of all the malignant umbilical tumors. The presence of umbilical subcutaneous nodule known as “Sister Joseph's nodule” has been commonly associated with intra-abdominal malignancy. The most common reported sites of origin for Sister Joseph's nodule are stomach (25%), ovary (12%), colorectal region (10%), and pancreas (7%) [2].

In a retrospective study of 145 patients with umbilical/paraumbilical hernia by Kenig et al., 23 patients (15.9%) were diagnosed with intra-abdominal malignancy. The logistic regression analysis demonstrated that the patient's age, preoperative symptoms, anemia, and weight loss were statistically significant risk factors that were associated with the presence of an intra-abdominal malignancy. The most common intra-abdominal malignancies are colorectal cancer (14 patients, 61%), pancreatic cancer (4 patients, 17.4%), malignant tumors of the adnexa (3 patients, 13%), and kidney cancer (2 patients, 8.7%) [3]. The College of American Pathologists recommends that the inguinal hernia sacs in adults and umbilical hernia sacs in children should be submitted to the pathology department for examination. Most often these specimens require only gross examination, but exceptions are made according to the pathologist's discretion [4].

There are recently published articles that question the utility of histologic evaluation of hernia sac specimens in the practice of cost-effective medicine [5, 6]. Wang et al. reported that, in their experience, malignancy in umbilical hernia is grossly not seen, and the diagnosis of malignancy was more frequent in umbilical hernias (1.2%) than in inguinal (0.4%) or femoral (0%) hernias [6]. The English literature search revealed 17 case reports of secondary malignancies found in umbilical hernia [7–17]. The most common malignancy was metastatic ovarian cancer (9 patients, 53%). The remaining malignancies were colon cancer, pancreatic cancer, peritoneal mesothelioma, peritoneal adenocarcinoma, primitive neuroectodermal tumor (PNET), extragonadal sex cord tumor with annular tubules (SCTAT), and cancer of unknown primary. In the prior published case report of metastatic pancreatic cancer to the hernia sac, the patient presented with ascites in addition to the umbilical hernia [7]. In our patient, there was no suspicion of malignancy until the histologic evaluation of the hernia sac, highlighting the importance of thorough microscopic evaluation of hernia sac specimens for the detection of occult malignancy.

By radiology, the use of intravenous contrast with computed tomography can help aid in the early detection of pancreatic lesions. Given our patient's history of chronic kidney disease, he underwent a prior noncontrast computed tomography scan four months earlier, which did not detect the pancreatic lesion. After the histologic diagnosis, the computed tomography scan with intravenous contrast detected the 5 cm hypoattenuating mass. The standard modality for the detection of pancreatic cancer with >90% accuracy is multidetector computed tomography. The use of intravenous contrast is essential, because pancreatic adenocarcinomas are hypovascular and have lower attenuation than healthy pancreatic parenchyma [18]. However, there are cases of isoattenuating pancreatic cancers, with reported prevalence of 11-14%, which poses a diagnostic challenge [18, 19].

In conclusion, the routine histologic evaluation of the hernia sacs is important in the diagnosis of occult malignancies, because lesions may not be grossly evident. In addition, the use of intravenous contrast with computed tomography can help aid in the early detection of pancreatic adenocarcinoma, because most lesions present as a hypoattenuating mass.
